# Cancer mortality differences among urban and rural residents in Lithuania

**DOI:** 10.1186/1471-2458-8-56

**Published:** 2008-02-12

**Authors:** Giedre Smailyte, Juozas Kurtinaitis

**Affiliations:** 1 Institute of Oncology, Vilnius University, Cancer Control and Prevention Center, Polocko 2, Vilnius, Lithuania

## Abstract

**Background:**

The aim of this study was to describe and to compare the cancer mortality rates in urban and rural residents in Lithuania.

**Methods:**

Cancer mortality has been studied using the materials of the Lithuanian cancer registry. For the period 1993–2004 age-standardized urban and rural population mortality rates (World standard) were calculated for all malignant neoplasm's and for stomach, colorectal, lung, prostate, breast and cervical cancers. The annual percentage change (APC) was calculated using log-linear regression model, two-sided Mantel-Haenzel test was used to evaluate differences in cancer mortality among rural and urban populations.

**Results:**

For males in rural population cancer mortality was higher than in urban (212.2 and 197.0 cases per 100000) and for females cancer mortality was higher in urban population (103.5 and 94.2 cases per 100000, p < 0.05). During the study period the age-standardized mortality rates decreased in both sexes in urban residents. The decreasing mortality trend in urban population was contributed by decline of the rates of lung and stomach cancer in male and breast, stomach and colorectal cancer in female. Mortality rates in both urban and rural population were increasing for prostate and cervical cancers.

**Conclusion:**

This study shows that large rural and urban inequalities in cancer mortality exist in Lithuania. The contrast between the health of residents in urban and rural areas invites researchers for research projects to develop, implement, and enhance cancer prevention and early detection intervention strategies for rural populations.

## Background

Cancer is a global problem that is particularly evident in economically developed, industrialised nations such as those of the European Union, where large proportions of the population are elderly and have the highest risk of cancer. The analysis of mortality trends is an important tool to monitor cancer control, and evaluate the outcomes of modifications in population lifestyle, environmental risks, and the effectiveness of health care. Examination of the trends in cancer mortality in Europe over the past 30 years has shown that, after long-term rises, age-standardised mortality from most common cancer sites has fallen in the EU since the late 1980s [1].

Cancer is the second frequent cause of death in Lithuania like in other European countries. Cancer mortality in Lithuania has been increasing year by year. However, the mortality rates of major cancer sites during the very last years showed a decreasing tendency [2]. Mortality features were studied in Lithuania [3-5]. Unfortunately, relatively little research has been published on cancer mortality with the aim to explain the link of mortality rates to socio-medical aspects. The aim of this study was to describe and to compare the cancer mortality rates in urban and rural residents in Lithuania.

## Methods

The data files of the Lithuanian cancer registry were used in the study. The mortality rates for all cancer sites, cancers of the stomach, colon and rectum, lung, breast, cervix and prostate were analysed for the period of 1993–2004.

The registration of deaths in Lithuania is based on the medical death certificates. The reliability of death certification in Lithuania meets high standards as the death certificates are signed by certifying physician. The main and underlying cause of death are reported on death certificate. The validity of the coding of the diagnosis is checked at the moment of the issue of the certificate and finally – at the Department of Statistics.

The population data used in the analysis were obtained from the Department of Statistics. In Lithuania there were two censuses in 1989 and 2001. The census data of the year 2001 shows that the total Lithuanian population decreased in size from 3,7 mln to 3,5 mln. In this study were used population estimates in five year age groups from 1993 to 2004 made after census in 2001 from the Department of Statistics.

Rural and urban populations were defined according to the population size – the settlements in the country less than 3000 of population are adjusted to rural. By the 2001, census data in Lithuania were 67% of the population were urban and 33% rural.

The age-standardised rates were calculated for both sexes. The standardisation was performed by direct method using the World standard population. The annual percentage change (APC) was calculated using log-linear regression model, two-sided Mantel-Haenzel test was used to evaluate differences in cancer mortality among rural and urban populations [6].

## Results

### All cancer sites

**Figure 1:**
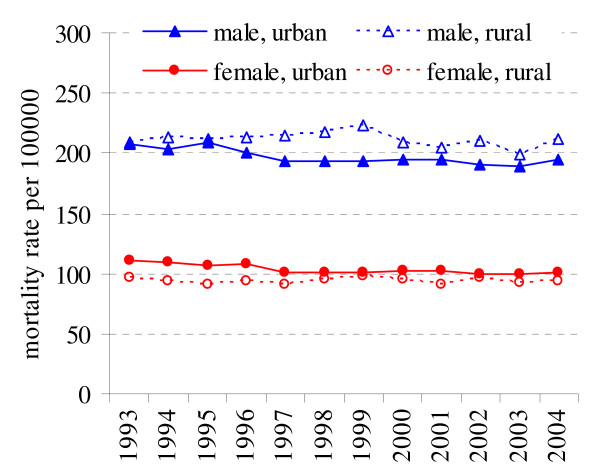
Cancer mortality in rural and urban population in 1993–2004 in Lithuania.

During the period from 1993 to 2004 in Lithuania the age-standardized mortality rates for male decreased from 205.3 per 100000 to 199.9 per 100000 and from 103.5 to 97.6 per 100000 for female. For males in rural population mortality was higher than in urban population (212.2 and 197.0 cases per 100000) and the observed differences were significant (p < 0.05). For females cancer mortality was higher in urban population (103.5 and 94.2 cases per 100000, p < 0.05). During the study period the age-standardized mortality rates decreased in both sexes in urban residents (Fig. 1). Between 1993 and 2004 in urban population mortality rates decreased among male by -0.73% and among female by -0.90% (Table 1). The mortality rates in rural population also have shown decreasing tendency in male (APC = -0.27%, p = 0.06) and were stable in female (APC = -0.08%).

**Table 1:** Mortality by cancer site, sex and place of residence in 1993–2004 in Lithuania.

			Number of deaths		Age standardized rates		
			
Site (ICD-10)	Sex	Residence	1993	2004	1993	2004	APC*
All sites (C00–C99)	Male	Urban	2460	2723	207.1	194.3	-0.73**
		Rural	1756	1840	209.1	211.6	-0.27
	Female	Urban	2080	2375	111.5	101.1	-0.90**
		Rural	1212	1210	97.4	93.4	-0.08
Lung (C33, C34)	Male	Urban	681	645	57.4	46.03	-2.06**
		Rural	598	544	72.5	62.8	-1.65*
	Female	Urban	132	139	6.8	5.8	-0.64
		Rural	72	77	5.2	5.3	0.15
Stomach (C16)	Male	Urban	364	289	30.6	20.5	-3.78**
		Rural	227	200	26.4	23.0	-2.06**
	Female	Urban	230	206	11.7	8.0	-2.83**
		Rural	176	112	13.1	8.1	-2.15**
Colon-rectum (C18–C21)	Male	Urban	231	301	19.7	20.7	0.17
		Rural	122	149	13.4	15.8	0.79
	Female	Urban	265	294	13.1	11.0	-1.85**
		Rural	144	158	9.3	9.5	-0.09
Breast (C50)	Female	Urban	390	414	22.3	19.7	-1.25**
		Rural	171	171	15.6	15.3	0.29
Cervix (C53	Female	Urban	137	150	7.6	7.5	0.69**
		Rural	84	109	8.9	12.2	2.34**
Prostate (C61)	Male	Urban	183	300	15.9	20.4	1.83**
		Rural	149	207	14.2	20.2	2.95**

* – APC – annual percentage change;

** – p < 0.05.

### Lung cancer

**Figure 2:**
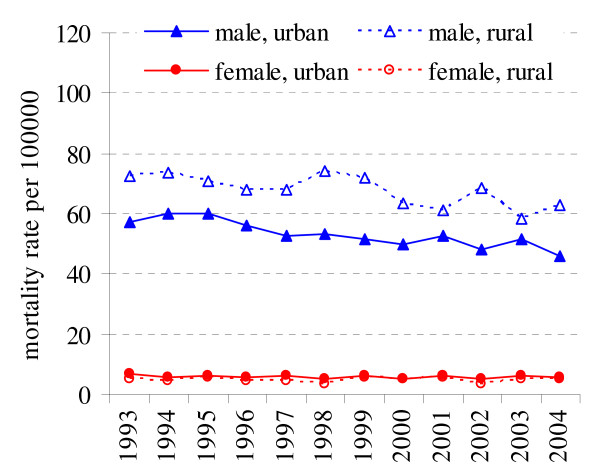
Lung cancer mortality in rural and urban population in 1993–2004 in Lithuania.

Lung cancer is the leading cancer among cancer related deaths in Lithuanian population (17.4% of all cancer related deaths in 2004, and 26.1% of cancer related deaths in males). Lung cancer mortality was significantly higher (p < 0.05) in rural population among males than in urban, while in females mortality rates were higher in urban than in rural population (p < 0.05) (Fig. 2). Mortality from lung cancer declined in the urban male population by -2.06% per year, whereas in rural population by -1.65% (Table 1). In females lung cancer deaths are less common, and no significant changes were observed for mortality rates for rural and urban residents (APC = 0.15%, p = 0.16 and APC = -0.64%, p = 0.40) during the study period.

### Breast cancer

**Figure 3:**
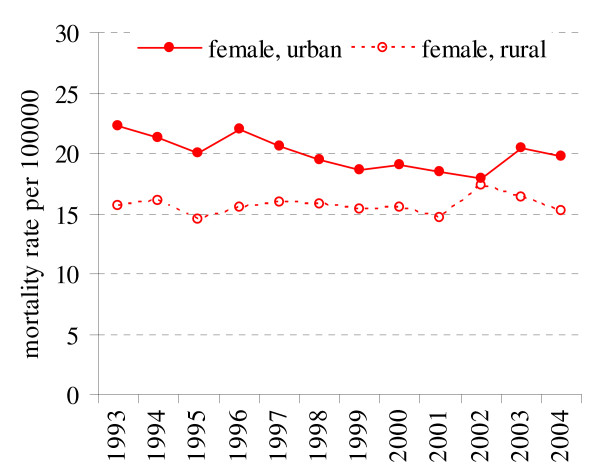
Breast cancer mortality in rural and urban population in 1993–2004 in Lithuania.

Breast cancer is the first cause of death among cancer related deaths in female population (16.6% in 2004). In the period of 1993–2004 there is a substantial decrease of breast cancer related deaths among urban resident (APC = -1.25%) (Fig. 3, Table 1). In female rural residents APC is estimated to be 0.29% (p = 0.18). Also, the overall breast cancer mortality was significantly higher among urban than rural females populations (p < 0.05).

### Stomach cancer

**Figure 4:**
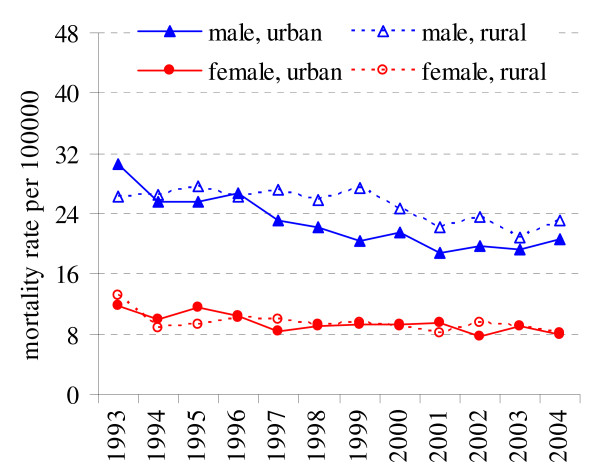
Stomach cancer mortality in rural and urban population in 1993–2004 in Lithuania.

During the period 1993–2004 the age-standardised mortality rates declined in both sexes. The significant differences in mortality rates were observed in male urban and rural residents (p < 0.05). The annual mortality decrease for stomach cancer in male urban residents was more rapid than in rural residents (APC = -3.78% and APC = -2.06%) (Fig. 4, Table 1). Annual percentage change is estimated as -2.15% in rural and -2,83% in urban population for female. No significant differences were found among female rural and urban stomach cancer mortality.

### Colorectal cancer

**Figure 5:**
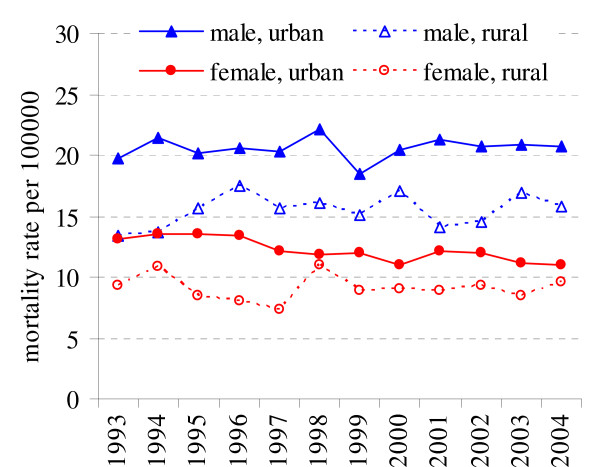
Colorectal cancer mortality in rural and urban population in 1993–2004 in Lithuania.

Significant higher mortality was observed for colorectal cancer in urban than in rural populations for both sexes (p < 0.05 and p < 0.05, male and female respectively). From 1993 the mortality rates for males were stable (APC = 0.79%, p = 0.79 and APC = 0.17%, p = 0.28 in rural and urban population) (Fig. 5, Table 1). In females colorectal cancer mortality in urban population decreased by -1.85% (p < 0.0001), while in rural females residents mortality rates were stable APC = -0.09% (p = 0.40).

### Cervical cancer

**Figure 6:**
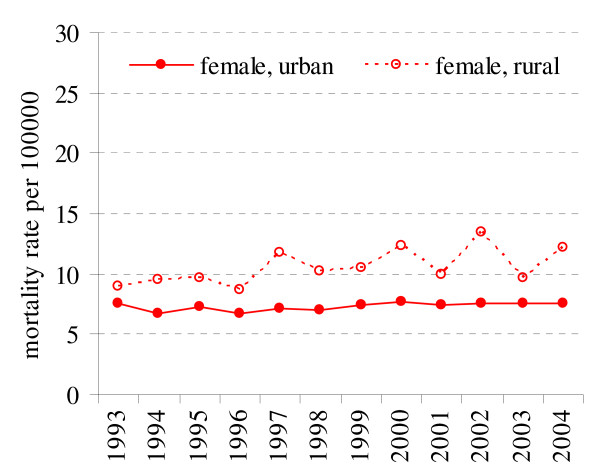
Cervical cancer mortality in rural and urban population in 1993–2004 in Lithuania.

Cervical cancer was the sixth most frequent cancer among the female. This cancer site was more common among female rural residents (p < 0.05) and showed an increase during the study period by 2.34% (Fig. 6, Table 1). In female urban residents the mortality rates were also increasing (APC = 0.69%, p = 0.02).

### Prostate cancer

**Figure 7:**
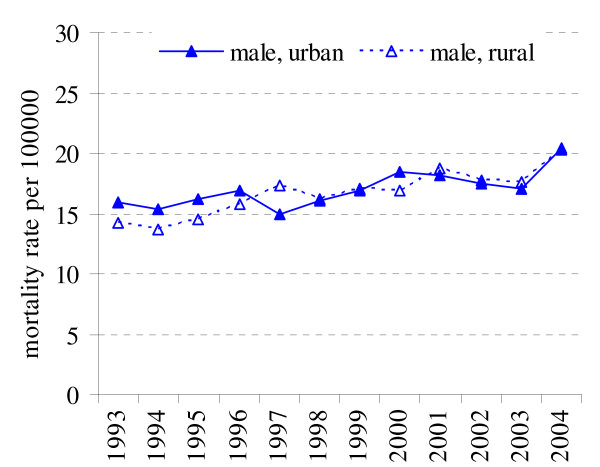
Prostate cancer mortality in rural and urban population in 1993–2004 in Lithuania.

In the year 2004 prostate cancer was the second most common cancer among male cancer related deaths (11.1% male cancer deaths). The prostate cancer mortality rates among urban and rural population were not different during the all study period (p > 0.05). During this period the substantial increase of number of deaths caused by prostate cancer was recorded for both rural and urban residents (APC = 1.83% and APC = 2.95%) (Fig. 7, Table 1).

## Discussion

The cancer mortality patterns reflect, to a certain extent, some basic characteristics of the society, such as life-style factors, population awareness, screening programmes, accessibility to health care and efficiency of health care system. In Lithuania during the period of 1993–2004 the age-standardised cancer mortality rates were decreasing in male and female urban population and were stable in rural residents. The overall decreasing cancer mortality trends seems to be stronger related to the decreasing mortality rates among urban residents, than in rural. The main contrast for males in mortality rates in urban and rural residents was observed for cancers of lung, stomach and colorectal, for females – for the breast, colorectal and cervical cancers. In general, cancer mortality trends in Lithuanian urban residents seems to be more favourable than in rural. There were observed more rapid mortality decrease (all cancer sites, lung and stomach cancer in males, colorectal and breast cancer in females) or increase was less pronounced than in rural population (prostate in males and cervical cancer in females). Major reductions in cancer mortality and morbidity are believed to be dependent on the widespread adoption of cancer prevention behaviours and use of early detection services.

The change in overall cancer mortality is the result of balance of trends for the different cancer sites. In the most of western European countries the total cancer mortality decreases because of decline in lung cancer mortality for males, the decline in stomach cancer for both sexes, and of cervical cancer for women, as well as some decline in breast and colorectal cancers [7]. In Lithuania decreasing mortality trend in urban population was contributed by decrease of the rates of lung and stomach cancer in male and breast, stomach and colorectal cancer in female. Mortality rates in both urban and rural population were increasing for prostate and cervical cancers.

In last decades the respiratory cancer deaths started to decline in other European countries [7]. Lung cancer mortality rates among men in Lithuania during the period from 1965 to 1994 showed an increase in cohorts born before 1945. In the younger generations, born after 1945, the risk declined [8]. The reduction in the number of lung cancer deaths among males was the major contributor to the overall decline in the number of cancer deaths in the Lithuania. Trends in lung cancer mortality mirror trends in smoking in the population. Thus, the progress in lung cancer mortality is due to the slow, but steady decline in smoking rates among men. Smoking rates appear to be dropping more slowly in rural residents. In contrast, the lack of progress in reducing smoking among females has led to stable lung cancer mortality among females.

Rates of mortality from breast cancer, the most common cancer in female in Lithuania recently have turned downward, probably at least in part because of better treatment. The fall in breast cancer mortality observed in most European countries over the last decade has to be attributed to earlier detection and improved treatment [9]. The mammography screening programme in Lithuania started in the 2005 and has not contributed to the observed decline in breast cancer mortality in the period from 1993 to 2004. Treatment improvements probably contributed to the mortality reduction because a favourable trend appeared before the introduction of the screening program; further improvements are expected as a consequence of the screening introduction [10].

Substantial decreases in mortality have occurred for stomach cancer in both sexes. The trend therefore corresponds to unplanned prevention through a changes in environmental factors occurring since the early 20th century. The exact causes of the decline of stomach cancer are not well understood, but must include improvements in diet, food storage (e.g., refrigeration) and, possibly, the decline of *Helicobacter pylori *infection [11]. The stomach cancer should still be considered in Lithuania as a major public health problem, despite the substantial decrease during last decades.

In Europe from 1997 to 2002 appreciable declines were observed in mortality from intestinal cancer in male (-1.6% per year), and in female (-2.5%) [1]. Colorectal cancer trends have been generally more favourable for female than for male. In Lithuania the mortality rates for males were stable and in females colorectal cancer mortality in urban population decreased, while in rural female residents mortality rates were stable. These temporal trends most probably reflect complicated interactions between early detection patterns and aetiological factors.

The cervical cancer mortality in Lithuania is the highest among the EU countries. The drop of the rates in cervical cancer mortality in Europe was largely due to screening. In Lithuania the national cervical cancer screening programme started in 2004 and has not yet contributed to the cervical cancer mortality rates. In Lithuania cervical cancer mortality rates were increasing in urban and rural female residents. The changes in the risk factors, such as in sexual behaviour and smoking habits, over the decades might partly explain trends in cervical cancer mortality.

Prostate cancer mortality from1997 to 2002 decreased in Europe by (-1.4%) per year [1]. Prostate cancer mortality increase was observed for both rural and urban residents in Lithuania.

Studies in other countries have identified significant disparities in stages of diagnosis between residents of rural and urban areas, with a greater incidence of late stage diagnosis generally found in rural areas. This has been attributed to limited access to clinics and hospitals with the advanced technology needed to detect cancer in the early stage and may need to travel great distances to receive care [12].

Most of the unfavourable cancer mortality patterns and trends in rural population are due to recognised, and hence largely avoidable, causes of cancer. Furthermore, there are high mortality rates for neoplasms related to inadequate screening, diagnosis and treatment. Rural residents tend to have lower income, higher poverty, less education. In rural settings no specialized services are available. The persistence of a wide variability in cancer mortality among urban and rural populations indicates that there is still wide area for prevention activities. The differences in cancer mortality among rural and urban residents in Lithuania are stimulating research to develop effective methods for cancer prevention and early detection services in rural populations.

## Conclusion

This study shows that large rural and urban inequalities in cancer mortality exist in Lithuania. The contrast between the health of residents in urban and rural areas invites researchers for research projects to develop, implement, and enhance cancer prevention and early detection intervention strategies for rural populations.

## Competing interests

The author(s) declare that they have no competing interests.

## Authors' contributions

GS was responsible for the development of intellectual content and the study design, statistical analyses, interpretation of the results and manuscript drafting. JK was responsible for interpretation of the results and the critical revisions of manuscript. Both authors read and approved the final manuscript.

## Pre-publication history

The pre-publication history for this paper can be accessed here:


